# Save Muscle Information–Unfiltered EEG Signal Helps Distinguish Sleep Stages

**DOI:** 10.3390/s20072024

**Published:** 2020-04-03

**Authors:** Gi-Ren Liu, Caroline Lustenberger, Yu-Lun Lo, Wen-Te Liu, Yuan-Chung Sheu, Hau-Tieng Wu

**Affiliations:** 1 Department of Mathematics, National Chen-Kung University, Tainan 701, Taiwan; girenliu@gmail.com; 2 Neural Control of Movement Lab, Institute of Human Movement Sciences and Sport, ETH Zurich, 8092 Zurich, Switzerland; caroline.lustenberger@hest.ethz.ch; 3 Department of Thoracic Medicine, Healthcare Center, Chang Gung Memorial Hospital, School of Medicine, Chang Gung University, New Taipei 33302, Taiwan; loyulun@hotmail.com; 4 Sleep Center, Shuang Ho Hospital, Taipei Medical University, New Taipei City 110, Taiwan; lion5835@gmail.com; 5 Department of Applied Mathematics, National Chiao Tung University, Hsinchu 30010, Taiwan; sheu@math.nctu.edu.tw; 6 Department of Mathematics and Department of Statistical Science, Duke University, 120 Science Dr. Durham, NC 27708, USA; 7 Mathematics Division, National Center for Theoretical Sciences, Taipei 106, Taiwan

**Keywords:** EEG, EMG, sleep stage classification, scattering transform

## Abstract

Based on the well-established biopotential theory, we hypothesize that the high frequency spectral information, like that higher than 100Hz, of the EEG signal recorded in the off-the-shelf EEG sensor contains muscle tone information. We show that an existing automatic sleep stage annotation algorithm can be improved by taking this information into account. This result suggests that if possible, we should sample the EEG signal with a high sampling rate, and preserve as much spectral information as possible.

## 1. Introduction

Electroencephalogram (EEG) is a widely used monitoring method to record brain activity in a non-invasive way. It has been extensively applied in various scientific and clinical setups. To date, it is still the gold standard method to define different sleep stages. In order to visually identify these stages, electrooculography (EOG, eye movement) and surface electromyography (EMG, muscle activation/tone) are further recommended, specifically to distinguish non-rapid eye-movement sleep (NREM) from REM sleep. Typically, in sleep laboratories all signals are recorded to provide an accurate sleep staging. The EEG signal is bandpass filtered at 0.3–35 Hz for the visualization purpose [1,2,3] according to the AASM criteria [4,5]. Furthermore, the raw data might be dumped from the commercial polysomnogram (PSG) and the raw data might have been filtered at the frequency range 0.3–35 Hz. Due to the advance of technology and focus on large-scale applications, there is an increasing number of mobile devices capable of recording EEG signal in in-home settings (e.g., single channel amplifiers on the forehead or in-ear/around ear applications) [6,7,8,9]. These mobile solutions might not include EMG and EOG information, and the raw data might be bandpassed for different purposes, like data storage or transmission.

According to the biopotential knowledge [10] about the spectral content of the recorded stimuli (action potentials) conducted by skeletal muscle fibers that contract (shorten) when stimulated, the spectral content above 25Hz of the EMG contains muscle tone information. Based on the normal muscle anatomy, while there is no muscle across the top of the head (epicarnial aponeuosis), we have the occipitofrontalis muscle that moves up the scalp and eyebrows, and several EEG leads are put on top of this muscle. In addition, while the amplitude of the EEG signal is of order 0.025–0.1 mV, the EMG signal is much larger and of order 0.1–100 mV. It is thus reasonable to infer that the high frequency spectral information (e.g., higher than 80Hz) of the EEG signal contains information related to surface EMG [11,12,13]. It is therefore interesting to ask if we can obtain sufficient muscle tone information from the high frequency range of the EEG signal, and use this information for any research or clinical purpose, if the sampling rate is high enough. Previous researchers have considered spectral power information around 80Hz in their sleep scoring algorithms and called 40–120Hz range as the *EMG band* [14,15], but to the best of our knowledge, generally information higher than 80Hz is less considered in sleep staging studies. Often, these frequencies are considered artificial noise in the sleep EEG and discarded. Yet, this “noisy” high-frequency bands might hold EMG related information that helps in sleep stage classification. To date, it is unclear whether this high-frequency content will add additional information to improve an existing sleep stage classification algorithm. This question is particularly important when we have only one EEG channel in a mobile health setup and not the standard montage in the lab that includes EMG for sleep stage differentiation. Clearly, unlike in the hospital setup, in this setup any single bit of information should be preserved and utilized.

In this paper, we want to illustrate that high frequency spectral information might capture EMG information and is therefore a valuable tool to better differentiate sleep stages. According to the sleep physiology [16,17], the EMG contains necessary information to distinguish REM and N1 (transition state in NREM sleep). Specifically, a normal subject is atonic during the REM stage, but the muscle tone is relative active during N1. Without the EMG, it might be challenging for a sleep expert, or a well-trained artificial intelligent (AI) system, to distinguish REM and N1. This fact has been well reflected in several state-of-the-art automatic sleep stage classification algorithms based on the single channel EEG signal [18,19,20,21,22]. As we can identify from those papers, the classification accuracy of N1 and REM is usually limited. We mention that the EEG signals in the commonly considered benchmark database in those papers, Sleep-EDF Database (Expanded) [23], are sampled at the sampling rate 100 Hz; that is, the spectral information higher than 50 Hz is not available. Motivated by the above-mentioned biopotential knowledge, in this report we hypothesize that we can improve an automatic sleep stage annotation system to obtain a more accurate N1 and REM classification if we keep the high frequency spectral information of the EEG signal.

## 2. Method and Material

### 2.1. Automatic Sleep Stage Annotation Algorithm

To show the usefulness of high frequency spectral information of the EEG, we consider a recently developed automatic sleep stage annotation algorithm from our group [22]. The algorithm is composed of three steps: preprocessing, unsupervised feature extraction, and learning. In the preprocessing step, we only apply the 60 Hz notch filter, and do not apply detrend or artifact rejection. We do not apply detrend or artifact rejection since the feature extraction algorithm summarized below automatically handles these issues.

**Figure 1:**
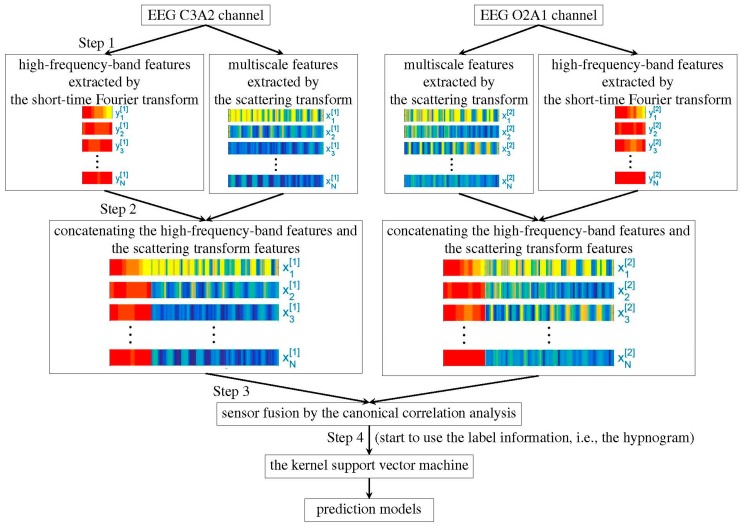
Flowchart of the proposed algorithm.

The feature extraction step is summarized in Figure 1. We firstly apply the recently developed scattering transform [24,25] to extract features from the EEG, filtered or unfiltered. This part of feature is obtained by iteratively applying the continuous wavelet transform and nonlinear modulus operators on a given EEG signal. It has been proved that the extracted features are stable to the time-warping deformation [24,25]. Moreover, they can effectively characterize self-similarity and intermittency properties of multiscale time series [26]. While the scattering transform is motivated by establishing a mathematical foundation of the convolutional neural network, it does not rely on the label information, so the feature extraction step in this algorithm is unsupervised. For unfiltered EEG signals, the authors further apply the short-time Fourier transform (STFT) to extract extra features from high frequency bands, including (35,80), (80,150), and (150,250) Hz. As indicated by Part 2 in Figure 1, the features obtained by the scattering transform and the short-time Fourier transform are merged together by the concatenation method. In view that each sleep stage has a correlation with the sleep stages before it, the temporal relationship is taken into account. Specifically, for each 30s epoch, we apply the scattering transform on a 90s EEG signal, which consists of the current 30s EEG signal and its previous 60s EEG signal. When we have two EEG channels, we apply the canonical correlation analysis (CCA) [27] to merge the heterogeneous information extracted from different channels and reduce their dimensions (indicated by Part 3 in Figure 1). Note that while we may consider modern dimension reduction methods, like the alternating diffusion maps [28], to simplify the discussion, we focus on the traditional CCA algorithm. Finally, we apply the well-established kernel support vector machine (SVM) [29] to learn the experts’ knowledge by classifying the CCA fused features (indicated by Part 4 in Figure 1).

We mention that the high frequency features associated with STFT was not considered in our previous work [22], otherwise the algorithm is the same. We refer readers with interest to that paper [22] for technical details. The code is implemented in MATLAB 2016a on a desktop PC with an Intel(R) Core(TM) i7-7820 3·6 GHz CPU and 64 GB of RAM, and is available via request. The scattering transform used in this study might be of independent interest, and readers having interest can download the generic code from www.di.ens.fr/data/software/scatnet.

### 2.2. Statistics

To evaluate the spectral relationship between EMG and EEG, for each considered frequency band and sleep stage, we construct a dataset collecting all spectral energies over the considered frequency band of all 30 s EEG segments that are labeled as the considered sleep stage from all ten subjects. A similar procedure is used to construct a database from the EMG segments. Then, we apply the Pearson correlation to study the linear relationship between these two datasets. To test the hypothesis that EMG and EEG’s spectral energies are uncorrelated, the permutation test [30,31]. With $10^{6}$ permutations is applied and we set the significant level to be 0.05. To account for the multiple test issue, the *p*-value is conservatively adjusted by the Bonferroni correction.

To evaluate how the high frequency spectral information impacts the performance of the automatic sleep stage annotation, we consider the *leave-one-subject-out* cross validation (LOSOCV). In the LOSOCV scheme, the database is divided into the training set and the validation set, and each set contains different subjects. As is discussed in our previous work [22], the LOSOCV scheme is challenged by the inter-individual variability and is close to the real-world clinical setup.

All performance measurements used in this paper are computed through the unnormalized confusion matrix $M\in R^{5\times 5}$ since we have 5 sleep stages to classify. For 1≤p,q≤5, the entry $M_{pq}$ represents the number of expert-assigned *p*-class epochs, which were predicted to the *q*-class. The precision (PR), recall (RE), overall accuracy (ACC), Cohen’s kappa (κ) coefficient and F1-score ($F1_{p}$) of the *p*-th class, where *p* = 1,…,5, are computed respectively from the confusion matrix. Finally, we evaluate the macro F1 score (Macro F1), which is the mean of 5 F1-scores associated with 5 sleep stages:

(1) $$\mathrm{Macro}\;F1=\frac{1}{5}\overset{5}{\underset{p=1}{\sum }}F1_{p}.$$

### 2.3. Database

The Institutional Review Board of Shuang Ho Hospital (SHH), Taipei Medical University, New Taipei City, Taiwan approved the study protocol (No. 101-4968A3). We consider the database from the sleep center at SHH collected from 2018/8/29 to 2019/4/26, where the subjects were suspected to have sleep apnea syndrome but ended up with a normal apnea-hypopnea index (AHI); that is, AHI is less than or equal to 5. A standard polysomnogram (PSG) study was performed with at least 6 h of sleep. The whole night signals were recorded by a biosignal amplifier system from Embla (NeuroLite, Belp, Switzerland). Standard signals, such as EEG signals (F3-A2, F4-A1, O1-A2, O2-A1, C3-A2 and C4-A1, where A1 and A2 are the reference lead located 1 cm above the mastoid process for the EEG recording), oral-nasal airflows, piezo-based abdominal and thoracic movement signals, electrocardiogram (ECG), electromyography (EMG), and photoplethysmography (PPG), were recorded. We focus on the EEG and EMG signals here, which are recorded at the sampling rate 500 Hz. We collected 10 subjects. The overnight sleep stages for all 30 s epochs, including Awake, REM, N1, N2 and N3, are provided by two sleep experts following the American Academy of Sleep Medicine (AASM) standard^5^. Among 10 subjects, there are 5 males and 5 females. For the 5 males, the age is 30.00 ± 7.84-year-old, the BMI is 24.09 ± 2.99 kg/$m^{2}$, and the AHI is 3.10 ± 1.57. For the 5 females, the age is 48.20 ± 12.15-year-old, the BMI is 21.95 ± 2.00 kg/$m^{2}$, and the AHI is 1.66 ± 1.57. The sleep recording time is 6.31 ± 0.64 h (Males: 6.06 ± 0.14 h, range 5.89–6.28 h; Females: 6.56 ± 0.87 h, range 5.91–7.96 h). Each subject’s EEG recording has two versions. In the first version, the high-frequency components in the EEG signals are filtered by a low-passed filter at 50 Hz, while in the second version the signal is not filtered.

## 3. Results

### 3.1. Relationship with EMG

The EEG signal in our database is sampled at the sampling rate 500 Hz, so theoretically we could inspect the spectral information up to 250 Hz. According to the biopotential theory, the spectral range of EMG is about 25–5000 Hz, while EEG is about 0.1–100 Hz. Therefore, to show how much muscle tone information is encoded in the EEG signal, we consider four different spectral ranges: (0.5, 15), (15, 35), (35, 80), and (80, 250). The first three spectral ranges are motivated by the EEG spectral range we are interested, while the fourth one is used to evaluate the EMG information encoded in the EEG. While there is no global consensus which high frequency spectral band is important, to the best of our knowledge, we further consider two finer spectral ranges, (80, 150) and (150, 250). There are in total 7556 30 s segments for analysis. The results of evaluating the relationship between EMG and central, occipital, and frontal EEGs are shown in Table 1, Table 2, and Table 3 respectively. The value of correlation is marked by * or ** if the p value is less than 0.05/36 = 0.0014, after Bonferroni correction, or $10^{-5}$, respectively. The higher the correlation coefficient, the more association we have between the EEG and EMG.

**Figure 2:**
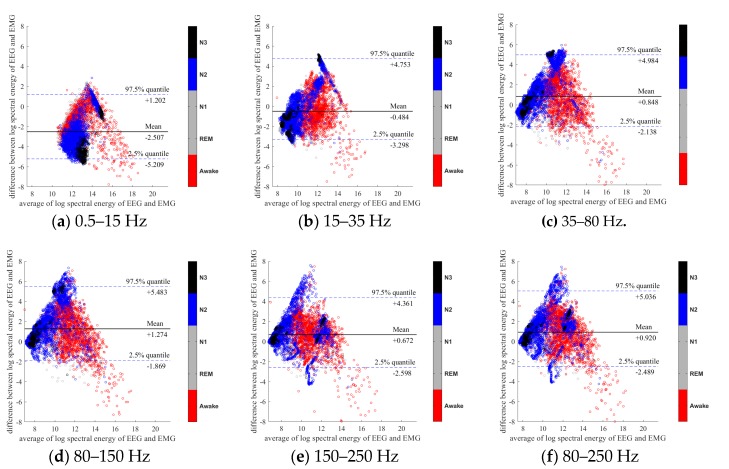
Bland-Altman plots of the logarithmic spectral energy of 30s (C3-A2) EEG and (Chin) EMG signals. To enhance the visualization, N1 and REM are plotted in the gray color in the background.

**Figure 3:**
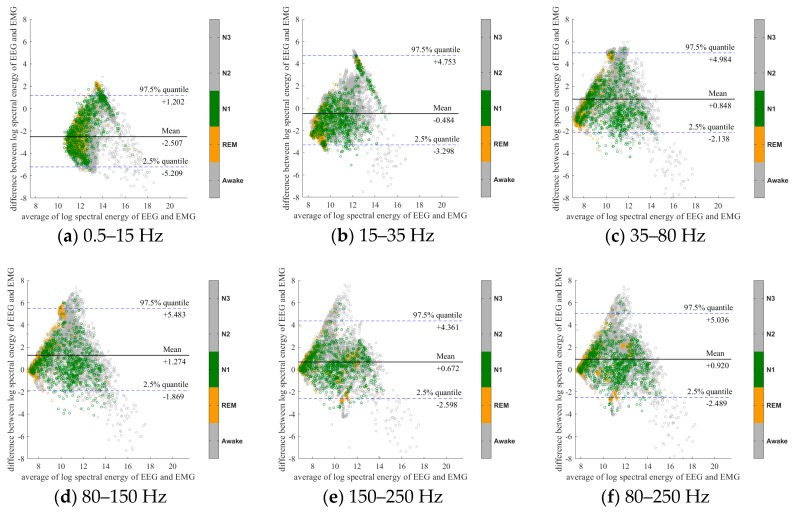
Bland-Altman plots of the logarithmic spectral energy of 30s (C3-A2) EEG and (Chin) EMG signals. To enhance the visualization, Awake, N2 and N3 are plotted in the gray color in the background.

**Figure 4:**
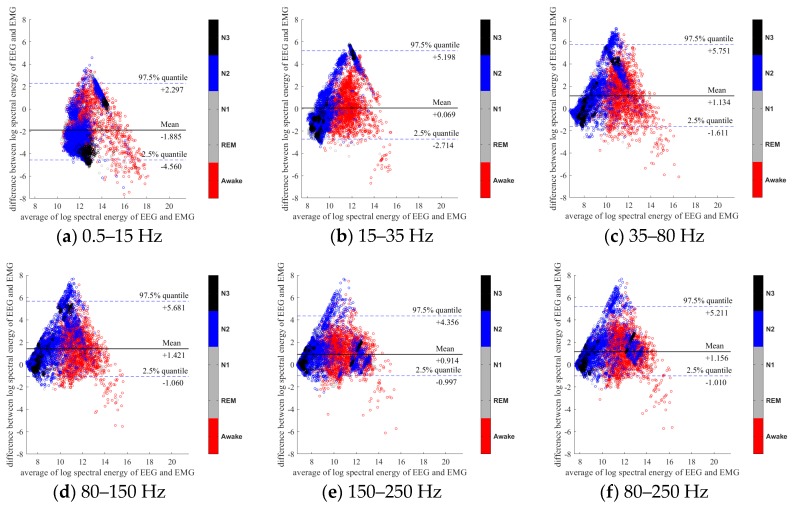
Bland-Altman plots of the logarithmic spectral energy of 30s (O2-A1) EEG and (Chin) EMG signals. To enhance the visualization, N1 and REM are plotted in the gray color in the background.

**Figure 5:**
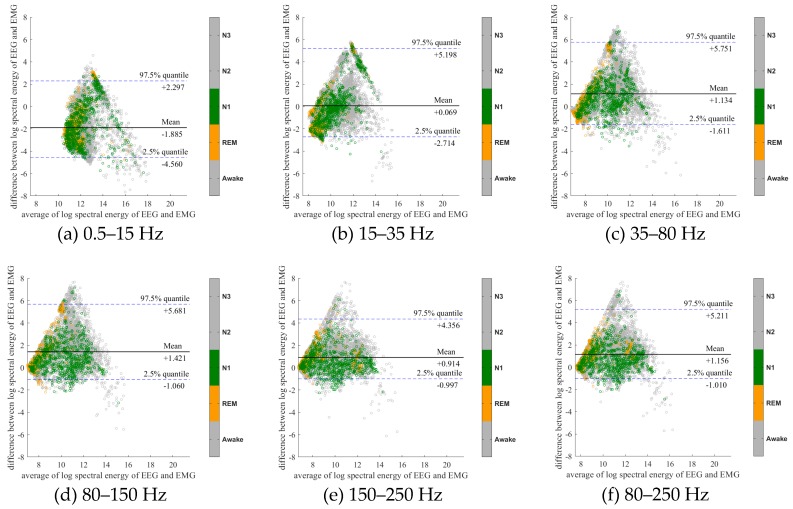
Bland-Altman plots of the logarithmic spectral energy of 30s (O2-A1) EEG and (Chin) EMG signals. To enhance the visualization, Awake, N2 and N3 are plotted in the gray color in the background.

**Figure 6:**
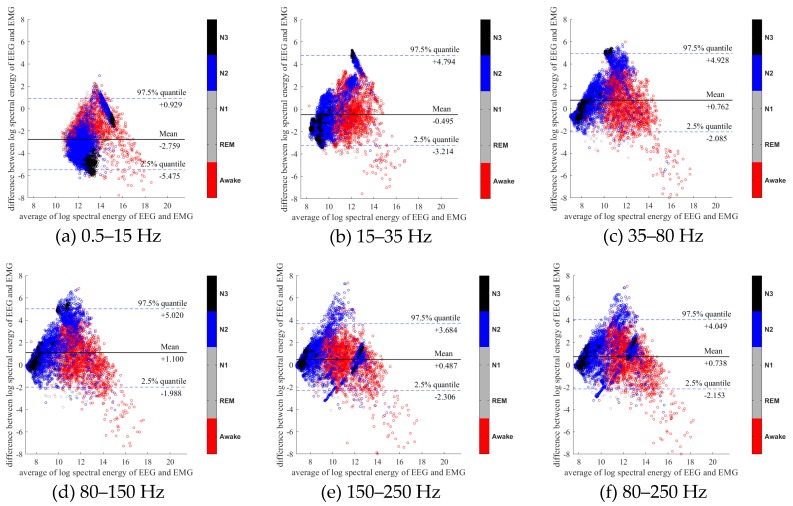
Bland-Altman plots of the logarithmic spectral energy of 30s (F3-A2) EEG and (Chin) EMG signals. To enhance the visualization, N1 and REM are plotted in the gray color in the background.

**Figure 7:**
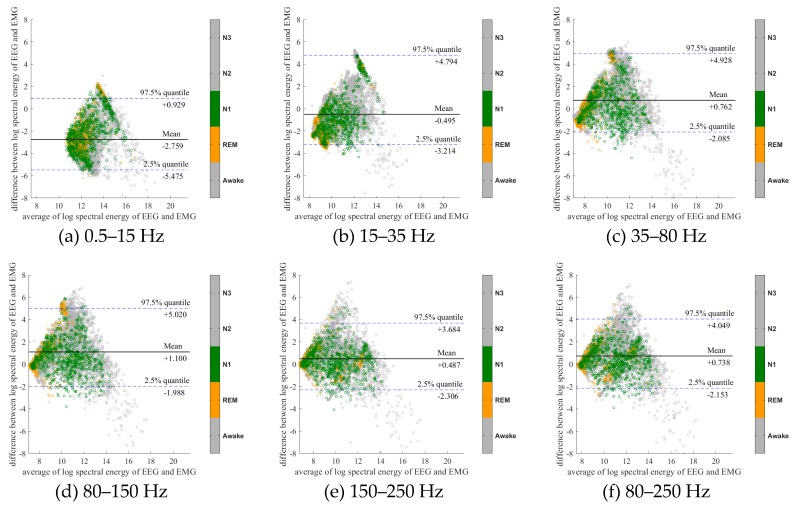
Bland-Altman plots of the logarithmic spectral energy of 30s (F3-A2) EEG and (Chin) EMG signals. To enhance the visualization, Awake, N2 and N3 are plotted in the gray color in the background.

To further evaluate the agreement between the EEG and EMG spectra, we plot the Bland-Altman plot [32] in Figure 2, Figure 3, Figure 4, Figure 5, Figure 6 and Figure 7. Because the number of Awake and N2 epochs is remarkably higher than that of REM and N1 epochs, the distribution of the REM and N1 dots will be hidden behind the Awake and N2 point clouds if all samples are plotted in the common window. Thus, to enhance the visualization, each figure is further divided into two subfigures, and the dots in both subfigures are the same except their colors. In the first (resp. second) subfigure, we highlight the distribution of Awake, N2, and N3 (resp. REM and N1) point clouds by plotting REM and N1 (resp. Awake, N2, and N3) points in gray as the background. We observed that usually the region covered by the REM and N1 dots is smaller than the region covered by Awake, N2, and N3 dots. On the other hand, note that the energy over the frequency range (150,250) Hz has the smallest 95% confidence interval (2.5% quantile, 97.5% quantile) in comparison with the energy over other high frequency bands, including (35,80), (80,150), and (80,250) Hz. The above results suggest that we can obtain a lot muscle tone information from the high frequency spectral information of EEG.

### 3.2. Automatic Sleep Stage Annotation Results

Table 4 is the confusion matrix obtained by applying the automatic annotation algorithm to low-pass-filtered EEG signals and Table 5 is the confusion matrix obtained by applying the algorithm to the original raw EEG signal. The increases of the main performance indices, including the overall accuracy (from 78.70% to 79.52%), macro F1 score (from 66.97% to 70.30%), and Cohen’s kappa (from 0.68 to 0.70), show that the high frequency spectral information in the unfiltered raw data is useful for the sleep stage classification. Furthermore, we have the following detailed observation.

- For the REM stage, Table 4 shows that 8% and 23% REM epochs were misclassified as N1 and N2 stages when the features extracted from low-pass-filtered EEG signals are used for classification. Table 5 shows that after taking high-frequency features extracted from the unfiltered raw data into account, the misclassification rate of REM epochs decreases (7% REM epochs were misclassified as N1 and 10% REM epochs were misclassified as N2).
- For the N1 stage, Table 4 shows that 17% and 29% N1 epochs are misclassified as REM and N2 stages, respectively. After taking high-frequency features extracted from the unfiltered raw data into account, Table 5 shows that the misclassification rate of N1 epochs slightly decreases.
- When the features extracted from the low-pass filtered EEG signals are used for classification, the F1 scores for REM and N1 stages are 66% and 29%, respectively. After taking high-frequency features extracted from the unfiltered raw data into account, the F1 scores for REM and N1 stages increase to 78% and 34%, respectively.

Note that Table 1, Table 2 and Table 3 show that the spectral energy of EEG and EMG signals within the frequency band above 100 Hz, particularly the band (150,250) Hz, has remarkably higher Pearson correlation on REM and N1 epochs in comparison with the correlation on Awake and N2 epochs. It means that we can obtain parts of EMG information from the unfiltered EEG signals during REM and N1 epochs. This may be the reason that the F1 scores of REM and N1 stages increase after taking high frequency spectral information in the unfiltered raw data into account.

## 4. Discussion and Conclusion

In this report, we show that high frequency EEG spectral information, particularly 80–250 Hz band, could be recycled to gain muscle tone information. By taking this information into account, it can help improve the existing automatic sleep stage annotation algorithm. This result suggests that if possible, particularly outside the hospital or when we do not have extensive monitoring equipment, we should sample the EEG signal with a higher sampling rate, and recycle as much high frequency spectral information as possible.

Physiologically, for a normal subject, the EEG signals during REM and N1 are indistinguishable. However, he/she is atonic during the REM stage, but not atonic during N1. To distinguish REM and N1, the sleep experts depend on reading EMG. Moreover, EMG provides information about sleep onset and macro-arousals during REM stage. As is shown in Section 3.1, the high frequency EEG spectrum encodes EMG information, particularly above 80Hz. This fact indicates that we can use the high frequency EEG spectral information as a surrogate of the muscle tone information. In Section 3.2, we show that the high frequency EEG spectral information does help improve the automatic annotation system. However, we shall mention that it is not clear from this study which information, like the size, shape or recruitment pattern of motor unit action potential, is encoded in the high frequency EEG spectrum. Also, as we could see, the EMG spectral information above 80 Hz could be better captured, while the EMG spectral information between 25–80 Hz is less well captured in the EEG signal, probably due to the interference of the brain activity. On the other hand, to the best of our knowledge, the relationship between the high frequency EEG spectral band and different sleep stages is less studied.

There are several possible applications of recycling the muscle tone information from the EEG signal. Recently, an increasing number of wearable biosignal amplifiers have been developed (e.g., around ear applications, wearable headbands) to obtain large-scale data and allow for in-field, long-term assessments of sleep [7,8,9]. One important aspect of these wearable devices is a non-obtrusive design and therefore a reduction of electrodes is desired. Specifically, electrodes around the chin for submental EMG assessment might be difficult to implement in a wearable design. Our results directly propose a solution to this problem by showing that much EMG information is already contained in the high-frequency EEG energy and can therefore partially replace EMG applications.

Another application of our findings could be in the operation room. Specifically, the bispectrum index (BIS) derived from the frontal EEG signal [33] is usually used in conjunction with the EMG to estimate the depth of anesthesia for the sake of minimizing the occurrence of intraoperative awareness. Similarly, during procedure sedation [34], the EMG could be taken into consideration to control the sedation level to reduce the side effect. In a recent paper [35], it is shown that the muscle tone information is directly related to BIS, which also supports the potential of recycling the muscle tone information from the EEG signal. To sum up, this suggests that in the situation that the BIS sensor is not available, we could count on a biopotential sensor on the frontal to obtain both EEG and EMG information, which enhance the healthcare quality. Outside the hospital, drowsiness detection for human control of motor vehicles or other facilities is an interesting application [36], since drowsiness is directly reflected in the muscle tone. It can also be applied to psychiatry related problems, like monitoring the stress release by the Yoga practice or meditation [37]. We will explore these interesting applications in future work.

## Figures and Tables

**Table 1:** Pearson correlation coefficient between EMG’s spectral energy and C3-A2 EEG’s spectral energy within various frequency bands during different sleep stages. The spectral range of each band is the same for the EEG and EMG signals. The dataset consisting of all 30s EMG and EEG segments from the ten subjects.

Frequency Bands	Sleep Stages					
	Awake	REM	N1	N2	N3	All
0.5–15	0.48 **	−0.12	0.16 **	0.07 *	0.23	0.21 **
15–35	0.25 **	0.20 **	0.45 **	0.24 **	0.00	0.42 **
35–80	0.34 **	0.28 **	0.60 **	0.44 **	0.08	0.56 **
80–150	0.42 **	0.35 **	0.65 **	0.46 **	0.22 *	0.60 **
150–250	0.57 **	0.81 **	0.77 **	0.65 **	0.93 **	0.73 **
80–250	0.48 **	0.70 **	0.72 **	0.58 **	0.90 **	0.68 **

**Table 2:** Pearson correlation coefficient between EMG’s spectral energy and O2-A1 EEG’s spectral energy within various frequency bands during different sleep stages. The spectral range of each band is the same for the EEG and EMG signals. The dataset consisting of all 30s EMG and EEG segments from the ten subjects.

Frequency Bands	Sleep Stages					
	Awake	REM	N1	N2	N3	All
0.5–15	0.43 **	−0.13	0.27 **	−0.14	−0.36	0.18 **
15–35	0.20 **	−0.07	0.40 **	−0.08	0.07	0.32 **
35–80	0.32 **	0.19 *	0.61 **	0.32 **	0.36 **	0.55 **
80–150	0.41 **	0.39 **	0.68 **	0.48 **	0.20 *	0.63 **
150–250	0.61 **	0.81 **	0.82 **	0.73 **	0.94 **	0.79 **
80–250	0.49 **	0.81 **	0.78 **	0.66 **	0.91 **	0.75 **

**Table 3:** Pearson correlation coefficient between EMG’s spectral energy and F3-A2 EEG’s spectral energy within various frequency bands during different sleep stages. The spectral range of each band is the same for the EEG and EMG signals. The dataset consisting of all 30s EMG and EEG segments from the ten subjects.

Frequency Bands	Sleep Stages					
	Awake	REM	N1	N2	N3	All
0.5–15	0.56 **	0.03	0.36 **	0.11 **	−0.19	0.28 **
15–35	0.26 **	0.28 **	0.39 **	0.19 **	−0.08	0.40 **
35–80	0.40 **	0.33 **	0.59 **	0.50 **	0.06	0.59 **
80–150	0.47 **	0.43 **	0.67 **	0.55 **	0.25 *	0.65 **
150–250	0.61 **	0.86 **	0.79 **	0.73 **	0.95 **	0.78 **
80–250	0.53 **	0.80 **	0.76 **	0.71 **	0.93 **	0.75 **

**Table 4:** Confusion matrix obtained from 10-fold leave-one-subject-out cross-validation on *band-pass filtered* EEG over 0.5-50 Hz signals extracted from C3-A2 and O2-A1 channels. The overall accuracy equals 78.70%, the macro F1 score equals 66.97% and Cohen’s kappa equals 0.6803. The precision (PR), recall (RE), and F1 score for each sleep stage are described in the last three columns.

	Predicted					Per-class Metrics		
	Awake	REM	N1	N2	N3	PR	RE	F1
**Awake (27%)**	1894 (91%)	26 (1%)	91 (4%)	67 (3%)	1 (0%)	83	91	87
**REM (9%)**	27 (4%)	470 (66%)	54 (8%)	162 (23%)	0 (0%)	66	66	66
**N1 (11%)**	258 (32%)	137 (17%)	181 (22%)	238 (29%)	2 (0%)	41	22	29
**N2 (48%)**	97 (3%)	83 (2%)	116 (3%)	3143 (88%)	152 (4%)	85	88	86
**N3 (5%)**	0 (0%)	0 (0%)	1 (0%)	97 (27%)	259 (73%)	63	73	67

**Table 5:** Confusion matrix obtained from 10-fold leave-one-subject-out cross-validation on *unfiltered* EEG signals extracted from C3-A2 and O2-A1 channels. The overall accuracy equals 79.52%, the macro F1 score equals 70.30% and Cohen’s kappa equal 0.6972. The precision (PR), recall (RE), and F1 score for each sleep stage are described in the last three columns.

	Predicted					Per-class Metrics		
	Awake	REM	N1	N2	N3	PR	RE	F1
**Awake (27%)**	1817 (87%)	36 (2%)	164 (8%)	62 (3%)	0 (0%)	83	87	85
**REM (9%)**	15 (2%)	576 (81%)	48 (7%)	74 (10%)	0 (0%)	76	81	78
**N1 (11%)**	262 (32%)	90 (11%)	249 (31%)	213 (26%)	2 (0%)	39	31	34
**N2 (48%)**	84 (2%)	54 (2%)	184 (5%)	3106 (86%)	163 (5%)	88	86	87
**N3 (5%)**	0 (0%)	0 (0%)	1 (0%)	95 (27%)	261 (73%)	61	73	67
